# 3D ultrasound file reading and coordinate transformations

**DOI:** 10.21105/joss.01063

**Published:** 2019-01-11

**Authors:** Pádraig T. Looney, Gordon N. Stevenson, Sally L. Collins

**Affiliations:** 1 Nuffield Department of Obstetrics and Gynaecology, University of Oxford, Level 3, Women’s Centre, John Radcliffe Hospital, Oxford; 2 School of Womens and Childrens Health, University of New South Wales, Randwick, NSW, Australia; 3 Fetal Medicine Unit, The Womens Centre, John Radcliffe Hospital Oxford

## Abstract

The Kretzfile format is used to store 3D ultrasound data, from GE Voluson ultrasound scanners. The geometry used in these hies is a toroidal coordinate system. Cartesian coordinates are required to allow application of advanced image libraries like ITK and scikit-image.

We present ITK transformation and utilities to convert Kretzfiles to cartesian coordinates. Previous work (SlicerHeart, 2017) has enabled the reading of kretz files and approximate coordinate transformations. This work will enable medical imaging researchers to investigate clinically 3D ultrasound.

## Tests

There are four tests included. A sample KretzFile is downloaded to the test directory and is used in the first three tests. The first test runs the executable KretzFileWriter and writes the image data, out to a Nifti format where the coordinates are in toroidal format. The second test runs the executable KretzConverter to output the image data, to the cartesian coordinates with a, voxel spacing of 0.6mm. The third test runs the executable KretzFileWriter to convert the output from the second test back into the toroidal coordinate system and saves the resulting image in Nifti format. These executables will allow researchers to analyes 3D ultrasound data in uncompressed Kretzfile format. By allowing the mapping back and forth from the toroidal coordinate system analysis can be performed in either coordinate system.

The toroidal planes through the volume are shown on the top row. The cartesian planes are shown on the bottom row.
